# DHN-YOLO: A Joint Detection Algorithm for Strawberries at Different Maturity Stages and Key Harvesting Points

**DOI:** 10.3390/plants14223439

**Published:** 2025-11-10

**Authors:** Hongrui Hao, Juan Xi, Jingyuan Dai, Guozheng Wang, Dayang Liu, Liangkuan Zhu

**Affiliations:** 1 College of Computer and Control Engineering, Northeast Forestry University, Harbin 150040, China; 13780832675@163.com (H.H.);; 2 Department of Mechanical and Electrical Engineering, Hebei Vocational University of Technology and Engineering, Xingtai 054000, China

**Keywords:** strawberry fruit, keypoint detection, ripeness detection, YOLO11-pose, deep learning

## Abstract

Strawberries are important cash crops. Traditional manual picking is costly and inefficient, while automated harvesting robots are hindered by field challenges like stem-leaf occlusion, fruit overlap, and appearance/maturity variations from lighting and viewing angles. To address the need for accurate cross-maturity fruit identification and keypoint detection, this study constructed a strawberry image dataset covering multiple varieties, ripening stages, and complex ridge-cultivation field conditions: MSRBerry. Based on the YOLO11-pose framework, we proposed DHN-YOLO with three key improvements: replacing the original C2PSA with the CDC module to enhance subtle feature capture and irregular shape adaptability; substituting C3K2 with C3H to strengthen multi-scale feature extraction and robustness to lighting-induced maturity/color variations; and upgrading the neck into a New-Neck via CA and dual-path fusion to reduce feature loss and improve critical region perception. These modifications enhanced feature quality while cutting parameters and accelerating inference. Experimental results showed DHN-YOLO achieved 87.3% precision, 88% recall, and 78.6% mAP@50:95 for strawberry detection (0.9%, 1.6%, 5% higher than YOLO11-pose), and 83%, 87.5%, 83.6% for keypoint detection (1.9%, 2.1%, 4.6% improvements). It also reached 71.6 FPS with 15 ms single-image inference. The overall performance of DHN-YOLO also surpasses other mainstream models such as YOLO13, YOLO10, DETR and so on. This demonstrates DHN-YOLO meets practical needs for robust strawberry and picking point detection in complex agricultural environments.

## 1. Introduction

Strawberries have become one of the world’s most popular berry fruits due to their unique taste and rich nutritional content, possessing extremely high economic value [1]. In China, strawberries are equally highly favored, and the country has emerged as the global leader in both cultivation area and production output [2]. At present, strawberry picking relies heavily on manual labor, which is not only inefficient but also faces labor shortages and cost increases as strawberry production increases and the number of people who devote themselves to agricultural production decreases [3,4]. Therefore, the development of automated strawberry harvesting robots represents a crucial strategy to address these pressing issues in the strawberry industry [5]. The first stage of automated strawberry picking involves using machine vision technology to accurately identify ripe strawberries and their picking locations [6,7]. However, in real-world conditions, strawberry appearance is influenced by ripeness level and variety, while issues such as shading and overlapping between fruits and foliage may occur. Furthermore, the requirement for fruit storage durability during long-distance transport necessitates precise sorting between fully ripe and ripe strawberries, posing challenges for accurately identifying strawberries at different maturity stages and harvest points [8,9]. We should also fully consider the computational capabilities of edge computing devices, making it essential to develop a vision algorithm that balances accuracy and speed [10].

Alongside the continuous advancement of artificial intelligence (AI) technology, deep learning has been widely applied in diverse domains of object detection, and the tasks of fruit identification and picking point localization are no exception to this trend. Yang et al. [11] proposed the PDSE-DETR model, an improvement over RT-DETR, to address the challenge of detecting strawberry ripeness in complex environments. This model reduces computational costs while enhancing accuracy. Li et al. [12] proposed an improved Transformer-based strawberry disease recognition method. They constructed a multi-class strawberry disease dataset comprising 5369 images of 12 common strawberry diseases. By employing multi-head self-attention and a Transformer based on spatial convolutional self-attention, the method achieved a disease recognition accuracy of 97.10%. Habaragamuwa et al. [13] utilized a region-based convolutional neural network (R-CNN) framework to detect ripe and unripe fruits of the “Akai Shizuku” strawberry variety under natural illumination conditions in a greenhouse environment. Yu et al. [14] applied Mask R-CNN to identify strawberry picking points in field environments.

By leveraging its region-based convolutional neural network architecture, the method enhanced the detection performance of strawberry picking points under complex conditions. The You-Only-Look-Once (YOLO) model proposed by Red et al. [15] has enabled object detection algorithms to enter a new phase with its faster speed and recognition capabilities. Zhou et al. [16] compared aerial imaging and ground-level imaging using YOLOv3 to evaluate recognition results across different stages of plant maturity. Zhan et al. [17] grounded their research on YOLOv4-tiny, achieving improved computational efficiency by reducing the number of network layers, modifying its network architecture, and replacing the original CSPNe module with two CSPNet modules characterized by fewer parameters and a simpler structure. This optimization thereby enabled real-time detection of strawberries. Yang et al. [18] proposed the LS-YOLOv8s model, which incorporates the LW-Swin Transformer module. For strawberry detection in complex field-growth environments, this model achieved a detection accuracy of 94.4% while maintaining a high detection speed. He et al. [7] proposed a two-stage deep learning framework that integrates an enhanced YOLOv8s with YOLOv5s-CLS. Specifically, the first stage involves an enhanced YOLOv8s model, incorporating C3x modules, an additional head network, and IOU-based techniques, for strawberry detection. Subsequently, the second stage employs YOLOv5s-CLS to perform maturity classification of strawberries, thereby determining their harvest readiness. This framework achieves high accuracy in strawberry detection, with classification accuracies reaching 100.0% for unripe strawberries and 95.0% for ripe ones.

While the aforementioned methods exhibit feasibility in fruit detection and picking point localization, their performance in strawberry recognition under complex real-world scenarios, such as severe occlusion and extreme lighting conditions, remains insufficient. Furthermore, strawberries have a delicate texture, necessitating precise picking point localization to mitigate damage; this requirement, however, remains incompletely addressed. Previous research has primarily focused on the binary classification of strawberry ripeness (i.e., “ripe” vs. “unripe”), neglecting the significant differences in transportation methods, storage requirements, and economic value between fully ripe and ripe strawberries in real-world production scenarios. Fully ripe strawberries have higher soluble sugar content, softer flesh, and thinner skin, making them more vulnerable to mechanical damage and microbial infection, which leads to spoilage. This necessitates cold-chain transportation throughout the supply chain, and limits their storage life to approximately 2 days. In contrast, mature strawberries have a firmer texture and greater resistance to pressure and dehydration, making them suitable for long-distance transport under conventional conditions. Their storage life extends to 3–5 days. Crucially, fully ripe strawberries typically command a 20–40% higher retail price than mature strawberries—an important economic distinction that directly impacts production profitability. Therefore, distinguishing between strawberries at the mature stage and those at the fully ripe stage is critical.

During the operation of automated harvesting equipment, detection models must not only maintain high accuracy but also ensure real-time performance. Although Transformer-based methods demonstrate strong feature representation capabilities, their high computational demands and heavy reliance on large-scale data make them unsuitable for real-time agricultural detection tasks. In contrast, while YOLOv11n-Pose achieves slightly lower detection accuracy than some Transformer models, its lightweight architecture, faster inference speed, and strong generalization ability for small- to medium-scale datasets make it more suitable for practical strawberry harvesting scenarios. Therefore, this study developed the DHC-YOLO model based on YOLOv11n-Pose, which achieves real-time recognition of strawberries at different maturity stages while maintaining high accuracy, enabling precise detection of key picking points for harvestable strawberries. The main contributions of this study are as follows:

Many publicly available strawberry datasets are collected under controlled environments, making it difficult to reflect complex conditions in actual cultivation settings such as fruit overlap, leaf shading, and uneven ripeness. To overcome limitations in image diversity, lighting, and occlusion, this study constructed a new strawberry dataset, MSRBerry, encompassing multiple varieties and growth stages. It includes di-verse environmental conditions such as occlusion, strong light, and shadows, providing a more representative data foundation for robust model training and evaluation in automated strawberry harvesting.

To address the limitations of the C2PSA module, which focuses on extracting global features and struggles with color variations and occlusion scenarios while overlooking small, irregular shape details—we designed the CDC module. By optimizing feature weights, this module enhances the model’s ability to capture fine-grained features and adapt to irregular shapes, enabling it to more effectively handle stem-leaf occlusion and color differences across varying maturity levels.

To more accurately capture features such as strawberry skin texture, we designed the C3H module to replace the C3K2 module. This module enhances multi-scale feature extraction through heterogeneous convolutions and deeply integrates multi-dimensional features, significantly improving the model’s robustness and detection accuracy under complex lighting conditions and multi-view scenarios.

The novel neck architecture is proposed by integrating the Channel Attention (CA) module with ConvTranspose2d layers into the original neck network, alongside designing two novel parallel feature fusion strategies “Multiplicative Fusion” and “Additive Fusion.” These enhancements not only strengthen feature representation capabilities and improve the efficiency of multi-scale feature interaction and fusion, enabling the model to handle occlusion and overlapping issues more effectively.

## 2. Discussion

The rapid advancement of deep learning has not only driven the technological evolution of complex neural networks themselves but also accelerated their application across multiple fields [19]. These technologies provide robust support for strawberry ripeness detection and automated harvesting. For example, Huang et al. [20] replaced the YOLOv5s backbone network with MobileNetV3 and introduced the Alpha-IOU loss function, thereby improving strawberry recognition accuracy and computational speed. Wang et al. [21] improved the YOLOv8+ model, enhancing the recognition accuracy of ripe/unripe strawberries. Existing research has primarily focused on the binary classification of strawberry ripeness (i.e., “ripe” vs. “unripe”), neglecting the significant differences in transportation methods, storage requirements, and economic value between fully ripe and ripe strawberries in real-world production scenarios. Furthermore, there is a lack of global models capable of integrating key point detection for identifying and harvesting strawberries across different ripeness stages. Furthermore, the complex real-world growing conditions—such as leaf occlusion, light variations, and fruit overlapping—pose additional challenges for the visual systems of automated harvesting robots.

To address these challenges, this study first constructed a strawberry dataset MSRBerry encompassing diverse real-world environmental conditions as the foundation for subsequent model training. The dataset includes scenarios such as strong direct sunlight, weak light shading, overlapping and interlaced fruits, and leaf occlusion, realistically simulating various interference factors that may arise during strawberry harvesting.

Based on the above analysis, this study proposes the DHN-YOLO model, an optimized variant of the YOLO11-pose architecture. This model accurately identifies strawberries at different maturity stages and efficiently detects key picking points. Test results indicate that for strawberry recognition across different maturity levels, the DHN-YOLO model achieves an accuracy of 87.3%, a recall of 88%, an mAP@50 of 91.8%, and an mAP@50:95 of 78.6%. For picking point detection, it achieves an accuracy of 83%, a recall of 87.5%, mAP@50 of 89.7%, and an mAP@50:95 of 83.6%, respectively. The model reaches a frame rate (FPS) of 71.6, with its single-image inference time below 15 milliseconds—fully meeting the real-time requirements of picking robots. Comparative experiments with multiple classical object detection algorithms and ablation studies of individual modules show that DHN-YOLO achieves significant accuracy improvement while maintaining real-time detection speed. This balance is attributed to the incorporation of the CDC module for capturing fine-grained strawberry features, the C3H module for enriching multi-scale feature extraction, and the New-Neck network for reducing feature loss and enhancing perception of key regions. Moreover, our model exhibits robust performance on external datasets. Finally, model visualizations reveal that even under intense lighting and overlapping occlusions, the DHN-YOLO model effectively focuses on strawberry regions while being minimally interfered with by irrelevant factors such as background clutter.

Future work will focus on expanding the dataset to include a wider range of agricultural scenarios and integrating multi-modal sensing techniques to further improve the recognition accuracy of strawberries at various maturity stages and their key picking points. Additionally, the model will be coupled with robotic manipulation systems to achieve full automation of the strawberry harvesting process.

## 3. Materials and Methods

### 3.1. MSRBerry Dataset

#### 3.1.1. Data Acquiring

**Figure 1:**
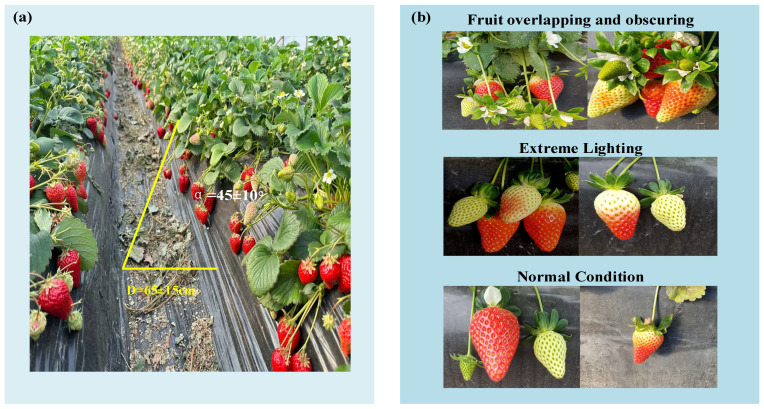
(**a**) Schematic diagram of camera position and angle during image acquisition; (**b**) Strawberry sample instances under different conditions in the dataset.

The dataset used in this study is a self-built dataset named MSRBerry Dataset (Multi-stage ridge-type strawberry dataset). It was collected from a strawberry cultivation base in Xiangfang District, Harbin City, Heilongjiang Province (Longitude: 126.626E, Latitude: 45.635N), which contains multi-variety strawberry greenhouses. The base adopts a ridge-planting cultivation pattern, and all dataset samples were obtained from ridge-planted strawberries. To closely replicate the visual environment encountered by harvesting robots during actual operations, this study employed a smartphone rear camera to capture images at a distance of approximately 50–80 cm from the ridge surface, positioned at an angle of about 45° relative to the ridge surface. This configuration simulates the downward-looking observation perspective of a harvesting robot, as illustrated in Figure 1a. Additionally, image acquisition was conducted under diverse field conditions, including complex scenarios such as strong light, weak light, fruit occlusion, and fruit overlap, as depicted in Figure 1b. To minimize data bias caused by different varieties, the MSRBerry dataset also includes samples of multiple strawberry varieties (“Sweet Charlie,” “Akihime,” “Myohyang”) at various stages of maturity. In total, 2066 images were selected to constitute the dataset, stored in PNG format with a resolution of 1024 × 768.

#### 3.1.2. Data Labeling and Augmentation

**Figure 2:**
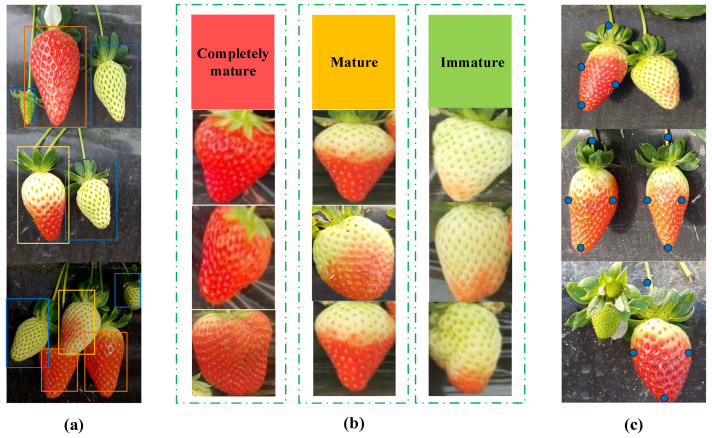
Examples of strawberry samples at different stages of ripeness, along with annotations of strawberry fruits and key harvesting points. (**a**) Annotated diagram of strawberries at different stages of ripeness; (**b**) Strawberry samples at different stages of ripeness; (**c**) Key points for strawberry harvesting.

This study classifies strawberry maturity into three grades based on the “Strawberry Grading Specifications” published by China’s Ministry of Agriculture, and the calibration of strawberries as shown in Figure 2a,b [22]. Strawberries in the green and white ripening stages—both unharvestable phases are defined as “unripe”; Strawberries in the late growth stage with 1/3 to 3/4 of their surface area covered in red are classified as “ripe,” suitable for storage or long-distance transport after harvest; Strawberries in the final growth stage that have enlarged to a vivid red color without any white-green mottling are classified as “fully ripe,” primarily intended for immediate sale after harvest. Given the soft texture of strawberries and their susceptibility to mechanical damage during harvesting, this study incorporated years of manual harvesting experience from fruit growers. For strawberries at the ripe and fully ripe stages with minimal shading, specific picking points were marked, and the calibration of strawberries as shown in Figure 2c. This approach facilitates the planning of mechanized harvesting paths, effectively avoiding vulnerable areas of the fruit and thereby reducing the risk of damage during the harvesting process. Each fruit was labeled with a bounding box defined by the minimum enclosing rectangle. Dataset annotation was performed using the Labelme tool, and the results were stored in .txt format. To further enhance the model’s generalization capability and robustness, data augmentation techniques (such as rotation, translation, and brightness adjustment) are applied to the original dataset to generate additional samples. After augmentation, the dataset expanded from 2066 to 5018 images. Finally, the dataset was split into training, validation, and test sets in an 8:1:1 ratio.

### 3.2. DHN-YOLO

#### 3.2.1. Architecture

The DHC-YOLO model proposed in this study adopts YOLOv11 as its base object detection framework, which is an optimized version of YOLOv8 developed by the Ultralytics team in 2024 [23]. The overall framework of YOLOv11consists of three core components: the backbone, neck, and head [24]. he backbone network extracts multi-level feature representations; the neck network integrates the SPPF (Spatial Pyramid Pooling–Fast) module to enhance adaptability to complex scenes; and the head network employs three output branches corresponding to large-, medium-, and small-scale objects [25].

**Figure 3:**
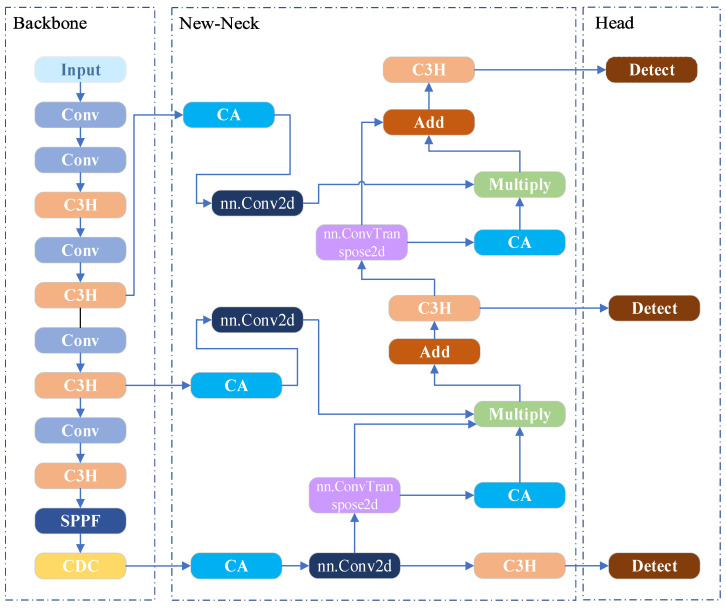
DHN-YOLO network architecture diagram.

At present, the YOLO11 model demonstrates outstanding performance in object detection. However, it still faces notable limitations in addressing the challenges of strawberry recognition and keypoint detection under complex field-growth conditions [25]. To overcome these issues and further enhance task-specific performance, this study builds upon YOLO11n-pose, a branch of YOLO11 designed for pose estimation and proposes the DHN-YOLO model. The network architecture of the proposed model is illustrated in Figure 3. First, within the backbone network, the position-aware self-attention mechanism in the C2PSA module is replaced with a cascaded group attention (CGA) mechanism, and the standard convolutions therein are substituted with deformable convolutions to construct a novel CDC module, thereby enhancing the model’s feature extraction capability in complex environments. By introducing a two-layer heterogeneous convolution (HetConv) to replace the standard convolution in the original C3K2 module and constructing the C3H module, the model not only achieves enhanced feature extraction efficiency but also becomes more sensitive to color variations under different lighting conditions. Finally, the Neck network incorporates a CA and a transposed convolutional layer (ConvTranspose2d), and employs a dual-path parallel feature fusion approach to construct the New-Neck network. The New-Neck network enables the model to better fuse multi-scale features and extract detailed color characteristics, enhancing the network’s recognition accuracy for ripe and fully ripe strawberries as well as the precision of identifying optimal picking points. Simultaneously, it significantly reduces computational load while ensuring real-time performance and accuracy.

#### 3.2.2. CDC

**Figure 4:**
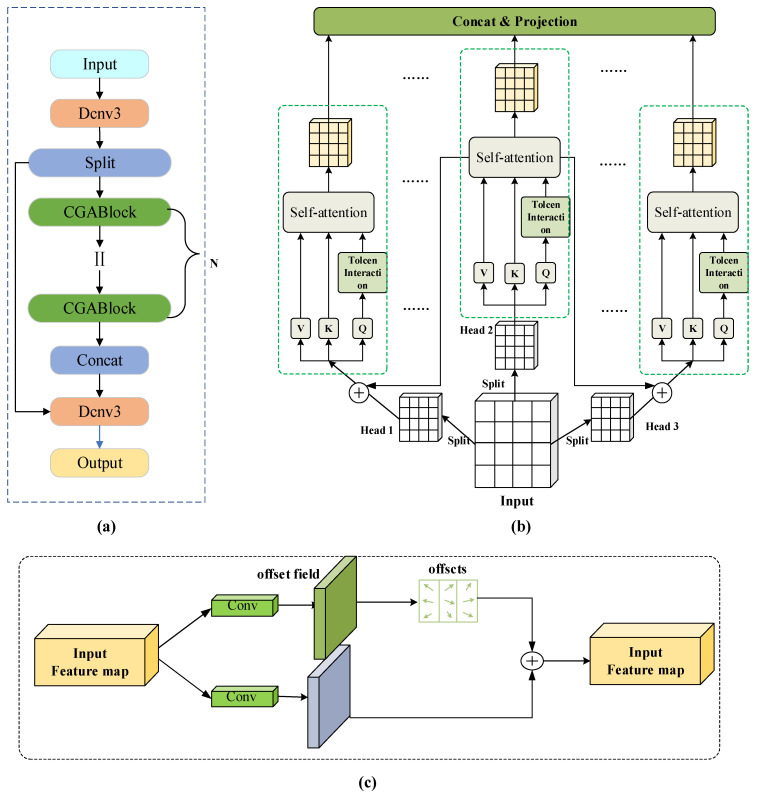
(**a**) CDC module architecture; (**b**) CGA module architecture; (**c**) DCNv3 module architecture.

In the context of multi-growth-stage strawberry recognition and picking point detection, strawberries exhibit notable variations in size and color across different developmental phases [26]. Furthermore, the complex growth environment, often cluttered with occlusions and irrelevant objects poses significant challenges to the accurate identification of strawberries and the precise localization of their optimal picking points. The original C2PSA module primarily leverages the Position-Sensitive Attention (PSA) mechanism for local feature modeling [27]. Although it can capture spatial features to a certain degree, it exhibits relatively limited capability in modeling fine-grained local details, inter-regional contextual relationships, global contextual features, and minute discriminative features. To mitigate these issues, we replaced the PSA mechanism and standard Conv (convolutional) components in the C2PSA module with the Cascaded Grouped Attention (CGA) mechanism and Deformable Convolution (DCNv3), respectively, thereby forming the C2DC module. The structure of this module is illustrated in Figure 4a. The CGA module [28] partitions the input features into distinct segments, each of which is processed by a dedicated attention head. Each head independently computes its self-attention mapping, after which the outputs from all heads are concatenated and linearly projected back to the original input dimension. This design substantially improves computational efficiency and facilitates the integration of both local and global information, thereby enabling the model to more effectively capture subtle variations on the fruit surface. The structural diagram is illustrated in Figure 4b. DCNv3 [29] addresses the limitations of fixed sampling points in traditional convolutions by incorporating learnable offset and grouping mechanisms. This enhancement allows the model to adapt more effectively to the complex growth environments of strawberries, as well as their irregular sizes and shapes. The corresponding structural diagram is presented in Figure 4c. The operation of DCNv3 is shown in Formula (1). Therefore, the C2DC module compensates for the shortcomings of the original C2PSA module in our strawberry identification and picking point detection task, thereby enhancing the model’s performance.

(1) $$y\left(u_{0}\right)=\sum_{g=1}^{G}\sum_{k=1}^{K}\alpha_{g}\beta_{gK}\delta_{g}(u_{0}{+u}_{k}+{∆u}_{gk})$$

Here, *u*_0_ denotes the current pixel, *G* represents the number of groups, *K* indicates the total number of sampling points. *α_gk_*∈*R^C^*^×^*^C^*^′^ (where *R* denotes the real number field), and *C*′ = *C/G* represents the group dimension. *β_gk_*∈*R^C^*^×^*^C^*^′^ denotes the softmax normalized modulation scalar at the *kth* sampling point in the gth group. *δ_gk_*∈*R^C^*^×^*^C^*^′^ represents the segmented input feature map (where *H* is the feature map height and *W* is the feature map width). *u_k_* denotes the *kth* predefined network sampling position, and Δ*u_gk_* is the offset corresponding to the *kth* network sampling position.

#### 3.2.3. C3H

**Figure 5:**
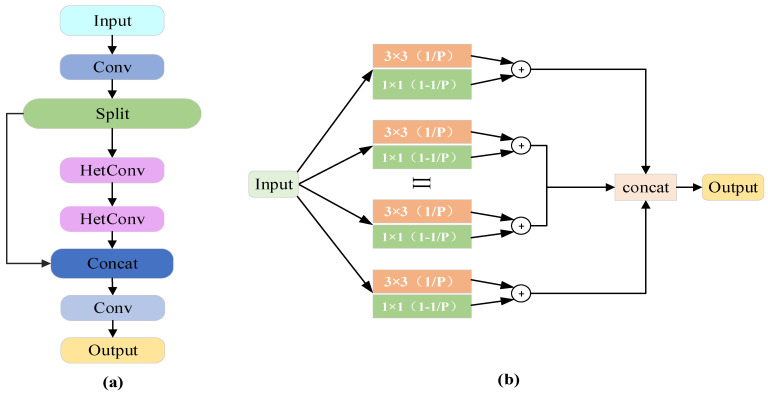
(**a**) C3H module structure diagram; (**b**) HetConv module structure diagram.

Under varying environmental lighting conditions and viewing angles, the ripeness, color, and morphology of strawberries may exhibit significant variations. Furthermore, the picking points are typically located at specific regions of the fruit. Consequently, accurate detection necessitates the integration of multi-scale features. To this end, we improved the C3K2 module that runs throughout the network by introducing two layers of HetConv to replace the Bottleneck/C3 modules, resulting in the C3H module, as illustrated in Figure 5a. HetConv [30] is a novel and highly efficient method that employs 3 × 3 and 1 × 1 convolutions. The 3 × 3 convolutions primarily capture spatially correlated information such as surface texture and calyx edges, while the 1 × 1 convolutions mainly preserve channel features like strawberry color. Finally, the features from both convolutional kernels are concatenated and fused, as illustrated in the structural diagram shown in Figure 5b. HetConv controls the ratio of the two kernels via parameter P (where 1/P represents the proportion of the 3 × 3 kernel, with the remainder allocated to the 1 × 1 kernel), as calculated by Equations (2) and (3). In this paper, P is set to 4.

(2) $${\mathrm{output}}_{i}={\mathrm{Conv}}_{3\times 3}\left(X_{3\times 3}\right)+{\mathrm{Conv}}_{1\times 1}(X_{1\times 1})$$

(3) $${\mathrm{output}}_{\mathrm{final}}=\mathrm{cancat}({\mathrm{output}}_{0},{\mathrm{output}}_{1},\ldots ,{\mathrm{output}}_{n})$$

*Conv*_3×3_ denotes a 3 × 3 convolution operation, *Conv*_1×1_ denotes a 1 × 1 convolution operation, and *X*_3×3_ and *X*_1×1_ represent the input data X divided into channel subsets according to specified rules. *output_i_* represents the output of each branch, while *output_final_* denotes the final output.

#### 3.2.4. New-Neck Network

**Figure 6:**
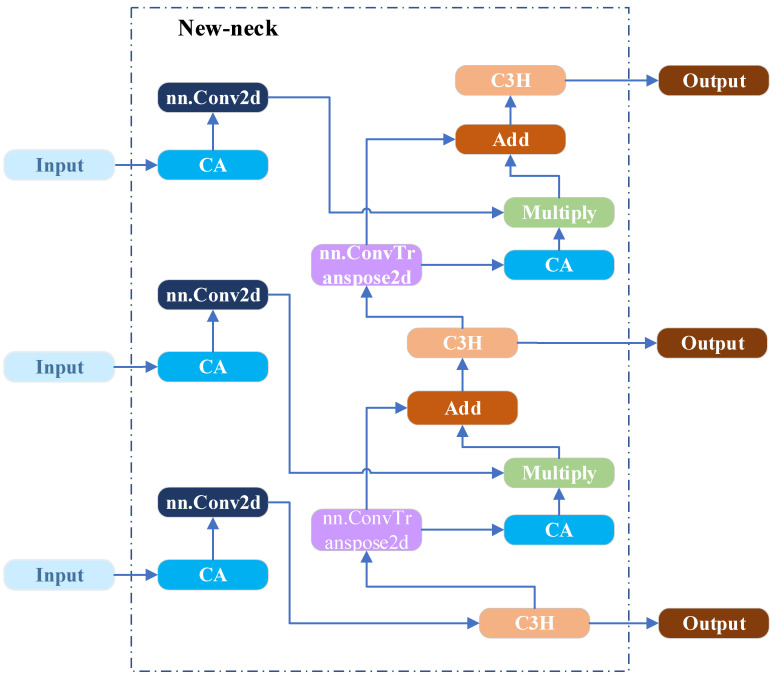
New-Neck network architecture diagram.

**Figure 7:**
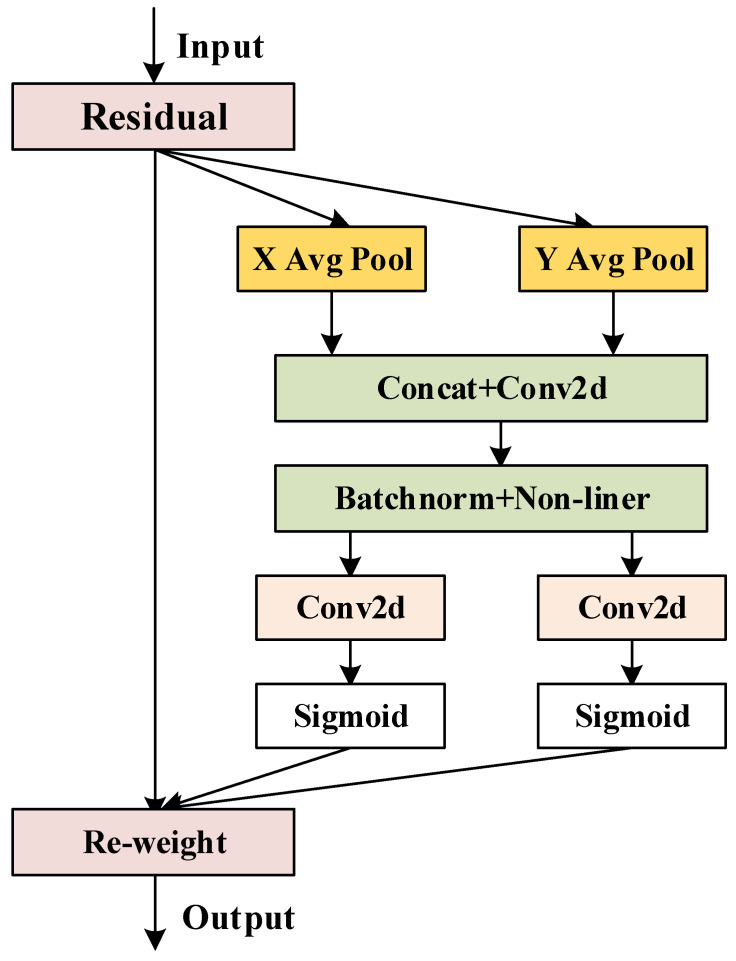
CA mechanism structure diagram.

In recent years, object detection models have been evolving toward high accuracy, real-time performance, and lightweight architectures to meet the real-time inference requirements of edge devices during field operations. This is of particular importance for strawberry recognition and the detection of picking key points. In addition, the original Neck network employs a simplistic feature fusion path and a single-channel concatenation operation, which struggle to effectively preserve small yet critical features, such as surface texture variations and stem edge details, leading to the loss of important feature information in this task. Additionally, since this architecture does not incorporate any attention mechanisms, the model exhibits poor sensitivity to critical regions such as small-scale immature fruits and picking points. This results in effective features being easily obscured by redundant or irrelevant information, such as leaf coverage. Additionally, the original network relies on stacking multiple convolutional layers for feature fusion, which leads to high computational complexity and reduced inference speed. We have made improvements to the Neck network, the structural diagram is shown in Figure 6. First, a CA was added to the multi-scale feature maps derived from the backbone network [31]. It captures global contextual information from the input features using average and max pooling layers, and subsequently generates channel attention weights through a Sigmoid function to suppress redundant or irrelevant channel information. The corresponding structure is illustrated in Figure 7. Before feature fusion, transposed convolutions (ConvTranspose2d) are applied for progressive upsampling, as defined in Equations (4) and (5), to align high-level features with low-level features. Subsequently, feature fusion is performed through two parallel paths: element-wise multiplication (Multiply) and element-wise addition (Add). This design preserves the integrity of the feature representation while emphasizing task-relevant features. These improvements effectively mitigate the feature loss and limited local context awareness in the original Neck network, thereby providing high-quality features and enhancing inference speed.

(4) $$H_{\mathrm{out}}=\left(H_{\mathrm{in}}-1\right)\times s-2p+k+\mathrm{outpu}t_{\mathrm{padding}}$$

(5) $$W_{\mathrm{out}}=\left(W_{\mathrm{in}}-1\right)\times s-2p+k+\mathrm{outpu}t_{\mathrm{padding}}$$

*H_in_* and *W_in_* represent the height and width of the input feature map; *H_out_* and *W_out_* represent the height and width of the output feature map; *s* denotes the stride; *p* denotes the padding; *K* denotes the size of the convolution kernel.

### 3.3. Experiments

#### 3.3.1. Experimental Settings

The experiments in this study were conducted on a Windows 11 operating system using PyCharm 2023, Python 3.9, PyTorch 1.12.1, and CUDA 11.6. The hardware configuration consisted of an Intel i7-13700KF CPU and an NVIDIA RTX 4090 D GPU with 24 GB of VRAM. No pre-trained models were utilized; all models were trained from scratch. The training parameters were set as follows: the input image size was 640 × 640, with a maximum of 200 iterations; the batch size was 16; the optimizer was stochastic gradient descent (SGD) with a cosine annealing strategy for dynamic learning rate adjustment. The initial learning rate was set to 0.01, the momentum factor to 0.937, and the weight decay factor to 0.0005. To ensure consistency, all models were trained on the same dataset under identical computational conditions.

#### 3.3.2. Evaluation Metrics

To evaluate the model’s performance, the following metrics were selected: precision (P), recall (R), mAP@50:95, number of parameters, and detection speed in frames per second (FPS). Here, mAP@50:95 denotes the mean average precision across all categories, with the Intersection over Union (IoU) threshold varying from 0.5 to 0.95 in increments of 0.05.

(6) $$P=\frac{\mathrm{TP}}{\mathrm{TP}+\mathrm{FP}}\times 100\%$$

(7) $$R=\frac{\mathrm{TP}}{\mathrm{TP}+\mathrm{FN}}\times 100\%$$

(8) $$\mathrm{AP}=\int_{0}^{1}P\left(R\right)\mathrm{dR}\times 100\%$$

(9) $$\mathrm{mAP}=\frac{1}{n}\sum_{i}^{n}\mathrm{AP}(i)\times 100\%$$

Among these, TP denotes true positive, FN denotes false negative, TN denotes true negative, and FP denotes false positive.

## 4. Experimental Results and Analysis

### 4.1. Ablation Experiment

To comprehensively evaluate the performance of the proposed model and validate the effectiveness and applicability of each proposed improvement module across different tasks, this study designed and conducted ablation experiments. The identification results for strawberry fruits at different stages are shown in Table 1. The key point detection results are shown in Table 2.

As observed from the experimental results, the independent integration of the C2DC module into YOLO11-Pose effectively enhances the model’s capability to perceive local fine-grained details and global contextual features in complex strawberry growth environments. Specifically, this improvement leads to accuracy gains of 0.8% in strawberry detection accuracy (for strawberries at different maturity stages) and 3.9% in keypoint localization accuracy, respectively. Correspondingly, the average precision (AP) for the two tasks increases by 0.9% (strawberry recognition) and 1.2% (keypoint detection). Notably, this enhancement was achieved without a significant increase in the number of parameters. When the C3H module and the improved New-Neck network are introduced separately, the C3H module significantly reduces the model parameter count (from 26.3M to 18.6M) while maintaining comparable detection accuracy. Meanwhile, the enhanced feature fusion mechanism in the improved New-Neck network effectively mitigates issues related to feature loss and insufficient perception of key regions (e.g., immature strawberries and picking points), resulting in average accuracy improvements of 0.4% for strawberry recognition and 2.4% for keypoint detection, respectively. These experimental results collectively validate the effectiveness of our proposed C2DC module, C3H module, and improved New-Neck network.

**Figure 8:**
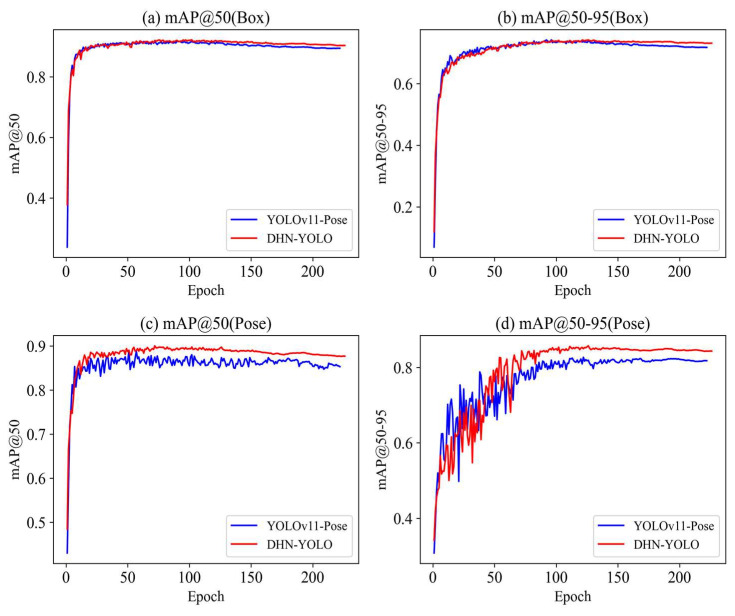
Comparison of average accuracy before and after improvement.

In the network models incorporating the two improved methods, both real-time performance and accuracy have been enhanced compared to the original YOLO11-Pose. The DHN-YOLO model, developed by integrating the three aforementioned improvement modules, outperforms the original YOLO11-Pose model in both core tasks. For strawberry recognition across different maturity stages, it achieves respective improvements of 1.1% in accuracy, 1.09% in recall, 0.9% in mAP@50 and 2.1% in mAP@50:95. For picking keypoint detection, the enhancements are even more notable: accuracy, recall, mAP@50 and mAP@50:95 increase by 1.9%, 1.9%, 1.4% and 4.3%, respectively. Additionally, the DHN-YOLO model balances performance with efficiency: its parameter count is reduced to 18.8M, while the single-image inference time is under 15 ms and the frame rate (FPS) reaches 71.6 fps. This efficiency level fully satisfies the real-time inference demands of strawberry-picking robots in field operations. The average precision comparisons between the improved models DHN-YOLO and YOLO11-pose are shown in Figure 8a,c. These results further validate the outstanding performance of DHN-YOLO in two core tasks: identifying strawberries at different maturity stages and detecting key points for strawberry picking.

### 4.2. Comparison Experiment

**Figure 9:**
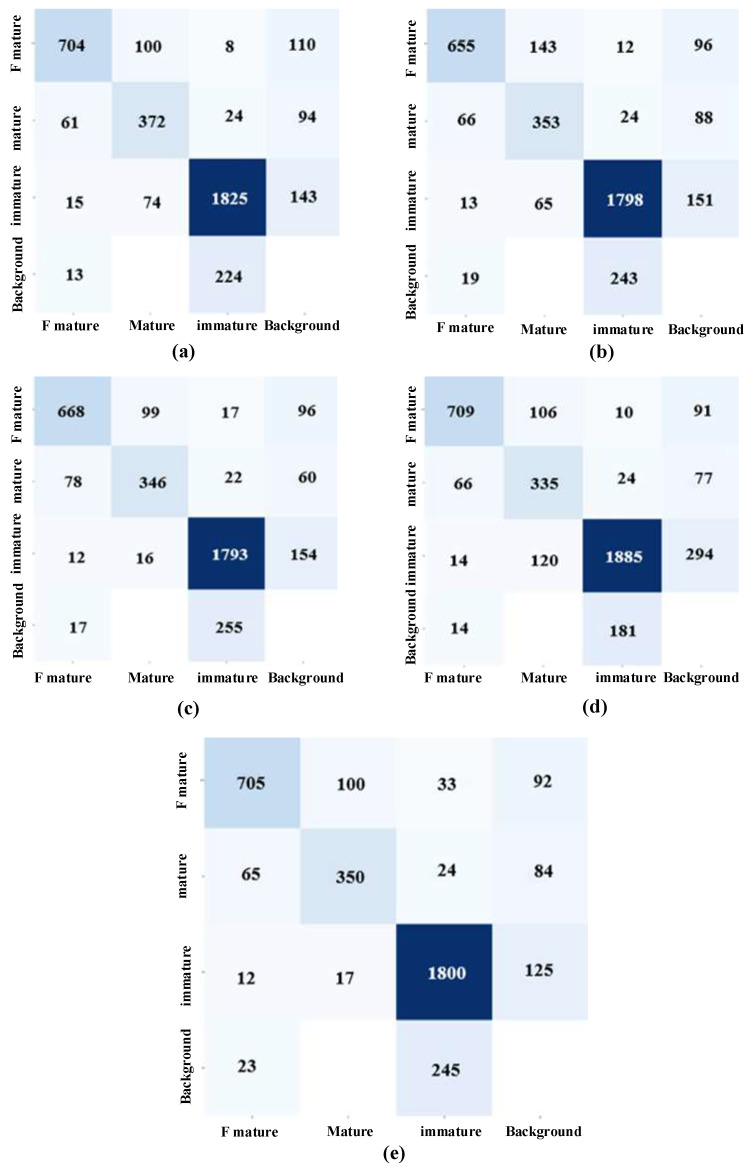
Confusion matrices for each model. (**a**) YOLO13-pose; (**b**) YOLO11-pose; (**c**) YOLO10-pose; (**d**) YOLO8-pose; (**e**) DHN-YOLO.

To further evaluate the performance of the proposed DHN-YOLO model in identifying strawberries at different maturity stages and detecting key picking points. When compared with other mainstream object detection networks (YOLOv13-Pose [32], YOLO11-Pose [33], YOLOv10-Pose [34], YOLOv8-Pose [35]), the results are shown in Table 3 and Table 4. n terms of strawberry recognition at different maturity levels, our DHN-YOLO performed well in accuracy and recall rates of 87.5% and 88%, respectively, and YOLOv13-pose performed outstandingly in average accuracy (mAP@50:95). For the mAP@50 metric, YOLOv13-Pose achieves 91.4%, while DHN-YOLO performs slightly better at 91.8%. The confusion matrices for each model are shown in Figure 9, where “F mature” denotes fully ripe strawberries, “Mature” denotes ripe strawberries, and “Immature” denotes unripe strawberries. It can be observed that misclassification between ripe and fully ripe strawberries is relatively prominent across all models, likely due to the minor differences in appearance characteristics such as shape and color between these two categories. However, recognition accuracy for unripe strawberries is generally high, indicating strong stability in distinguishing samples from early maturity stages. It is also evident that DHC-YOLO not only significantly reduces confusion between ripe and fully ripe strawberries but also effectively suppresses background interference, yielding more stable and balanced recognition results across all categories. Overall, DHC-YOLO demonstrated optimal performance in strawberry ripeness detection, combining high recognition accuracy with outstanding generalization capabilities. In the task of strawberry picking keypoint detection, the DHN-YOLO model achieves a mean average precision (mAP@50:95) that is 6.4% higher than YOLOv13-Pose, 4.6% higher than YOLO11-Pose, 2.3% higher than YOLOv10-Pose, and 9.5% higher than YOLOv8-Pose, respectively. Furthermore, it also exhibits clear advantages in accuracy and recall over most of the compared mainstream pose estimation models. Regarding inference speed, YOLOv10-Pose achieves the fastest inference performance at 80.7 FPS, followed by YOLOv8-Pose, which reaches an inference speed of 74.8 FPS. However, the performance of these two models in other key metrics specifically accuracy, recall, mAP@50, and mAP@50:95 for strawberry recognition and picking keypoint detection is relatively underwhelming. Our DHN-YOLO not only ensures accuracy for our tasks but also achieves 71.6 FPS, fully meeting the real-time needs of strawberry-picking robots. Therefore, based on a comprehensive comparison of key metrics (e.g., accuracy, mAP, inference speed, and parameter count), our DHN-YOLO is more suitable for the practical joint detection task of strawberry maturity recognition and optimal picking point detection in complex field environments.

**Figure 10:**
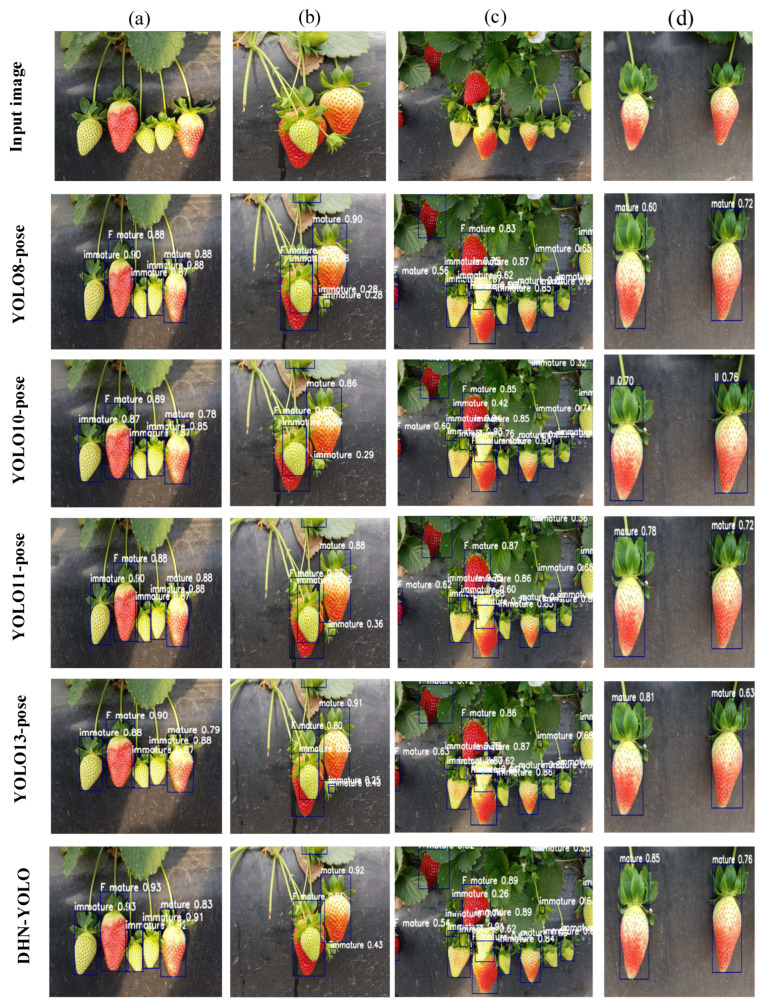
Visual comparison of different models in detecting key points for strawberry recognition and picking at various maturity stages. (**a**–**d**) show strawberry images in complex environments, including strong illumination, shadows, and overlapping fruits.

To validate the DHN-YOLO model’s ability to recognize maturity levels and detect key points in complex environments, the trained model was applied to identify strawberry fruits at different stages of maturity. The detection results are shown in Figure 10. Our DHN-YOLO model demonstrates the best performance, achieving recognition confidence levels of 0.6 to 0.9 or higher under extreme lighting conditions and 0.6 to 0.89 or higher when fruits are partially obscured. This indicates our model can effectively identify strawberries across various ripeness stages and pinpoint optimal harvest timing.

### 4.3. Cross-Dataset Validation

To further evaluate the model’s generalization capability, we employed the publicly available strawberry dataset by Alessandra Tafuro et al. [36] to validate the DHN-YOLO model trained on our proprietary dataset. This dataset encompasses strawberry samples across three ripeness stages unripe, partially ripe, and fully ripe and includes complex scenarios from various practical cultivation settings. Experimental results demonstrate that for strawberry recognition across different maturity stages on external datasets, DHN-YOLO achieves a precision (P) of 86%, a recall (R) of 88.2%, an mAP@50 of 92.2%, and an mAP@50:95 of 82.2%. For the keypoint detection task, the corresponding metric values are 85.8%, 86%, 93.5%, and 84.5%, respectively—all reflecting robust performance. This demonstrates that the DHN-YOLO model maintains excellent recognition and detection capabilities when confronted with cross-domain data, exhibiting strong generalization capabilities. To further assess the model’s robustness under complex real-world conditions, representative test samples exhibiting abnormal illumination, occlusions among fruits and leaves, and strawberries at various ripening stages were selected from the test dataset. The experimental results indicate that DHN-YOLO achieves a precision of 82.4%, a recall of 84.9%, an mAP@50 of 88.6%, and an mAP@50:95 of 72.6% for strawberry detection. For the keypoint detection task, the corresponding values reach 80.4%, 84.5%, 85.8%, and 80.5%, respectively.

These findings demonstrate that DHN-YOLO maintains superior detection accuracy and stability when facing challenges such as occlusion and lighting variation, confirming its robustness and efficiency in complex agricultural environments.

### 4.4. Visualization Analysis

**Figure 11:**
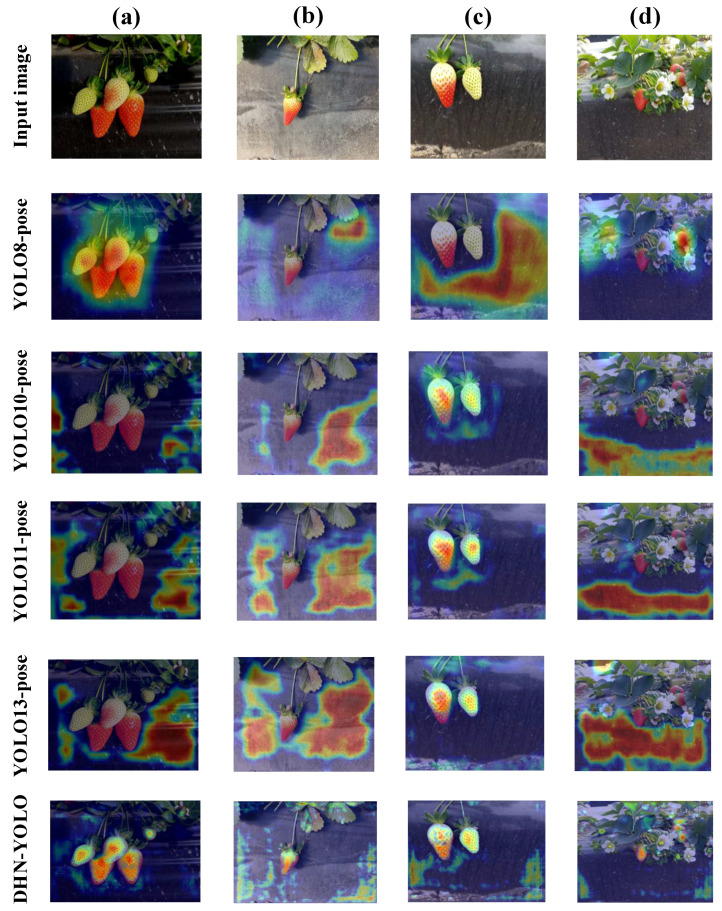
Comparison of Grad-CAM visualizations between YOLO8-pose, YOLO10-pose, YOLO11-pose, YOLO13-pose, and DCN-YOLO: (**a**) Low-light conditions, (**b**) Single fruit with significant background interference, (**c**) High-light conditions, and (**d**) Occlusion conditions.

To address the poor interpretability of deep learning models in strawberry maturity recognition and picking keypoint detection, this study employs Gradient-Weighted Class Activation Maps (Grad-CAM) to visually analyze the DHN-YOLO model. Grad-CAM [37] provides an intuitive representation of the regions the model attends to during inference. In the object detection process, Grad-CAM extracts critical feature cues by computing the gradient of the input image and converting them into a heatmap, thereby highlighting regions of high feature density that the model focuses on. The visualization results are shown in Figure 11, which includes five rows of Grad-CAM heatmaps corresponding to the models YOLO8-pose, YOLO10-pose, YOLO11-pose, YOLO13-pose, and the proposed DHN-YOLO. In the heatmaps, darker colors indicate stronger model attention [38]. Input images (a), (b), (c), and (d) correspond to strawberries under four different conditions: low light with multiple berries, a single strawberry, strong light, and an obstructed view, respectively. As shown in Figure 11, compared to previous YOLO detection algorithms, the proposed DHN-YOLO exhibits a stronger ability to accurately focus on strawberry target regions while minimizing interference from irrelevant factors such as background leaves and soil. Even under challenging conditions, such as strong illumination or fruit occlusion by foliage, DHN-YOLO consistently maintains superior focus on the strawberry subject. These findings confirm that DHN-YOLO can more effectively mine, extract, and learn key feature information from strawberry regions. It not only enhances feature aggregation and attention toward the target strawberries but also effectively suppresses interference from redundant background information.

### 4.5. Model Deployment

**Figure 12:**
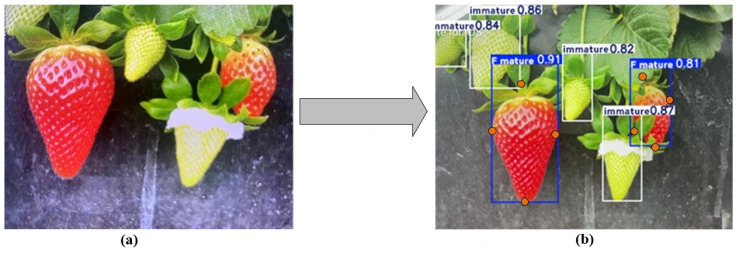
Real-time detection results after deploying DHN-YOLO: (**a**) Strawberry ripeness and key point detection and (**b**) Detection results.

To evaluate the real-time detection capability and edge-device inference performance of the proposed DHN-YOLO model, we deployed it on an ASUS TUF Gaming F15 FX507 laptop equipped with an Intel^®^ Core™ i7-13700H CPU and NVIDIA GeForce RTX 4070 GPU, using a smartphone as the image acquisition device to establish a portable mobile detection solution. Experimental results demonstrate that the model achieves an average inference time of approximately 11 ms per frame, corresponding to a frame rate of 90 FPS, thereby fully validating the real-time detection potential of DHN-YOLO. The post-deployment performance is illustrated in Figure 12, where the model accurately recognizes strawberries at different ripeness stages and precisely localizes optimal picking points, even under conditions of partial occlusion. Looking ahead, future work will focus on deploying the model to edge devices such as ground-based harvesting robots, enabling precise ripeness identification and automated harvesting, and ultimately facilitating its practical implementation in agricultural production.

## 5. Conclusions

To address practical detection challenges in complex strawberry harvesting scenarios, this study first constructed a strawberry dataset encompassing various real-world disturbances such as direct strong light, weak light occlusion, overlapping fruits, and leaf obstruction. Building upon this foundation, the DHN-YOLO joint detection model was proposed for identifying strawberries at different maturity stages and detecting key picking points. Compared to existing deep learning methods such as YOLO13-pose, YOLO11-pose, YOLO10-pose, YOLO8-pose, and DETR, the proposed DHN-YOLO demonstrates significant advantages in accuracy, robustness, and real-time performance. The CDC module enhances fine-grained feature extraction capabilities, while the C3H module strengthens multi-scale feature learning, enabling robust recognition under varying lighting and viewpoint conditions. Additionally, the innovative New-Neck architecture reduces computational complexity by integrating computer vision with dual-path fusion techniques, all while maintaining detection accuracy. Collectively, these improvements enable the model to consistently deliver strong performance across diverse and complex environments. On the self-built dataset, the strawberry maturity classification task achieves 87.3% accuracy, 88% recall, 91.8% mAP@50, and 78.6% mAP@50:95; For the picking point detection task, accuracy reached 83%, recall reached 87.5%,89.7% mAP@50, and mAP@50:95 reached 83.6%, with single-image inference time under 15 ms. Performance metrics across various tasks showed varying degrees of improvement. Additionally, the model demonstrated outstanding multi-task performance on public datasets. Ablation studies and comparative experiments further validated the effectiveness of each module and the overall performance advantages of the DHN-YOLO model. In summary, the DHN-YOLO model efficiently and accurately detects strawberries at different maturity stages and their key picking points in complex environments. It meets the practical application requirements for strawberry picking robot vision systems and provides reliable technical support for the visual perception module of automated strawberry harvesting equipment.

## Figures and Tables

**Table 1:** Results of strawberry fruit recognition and dissolution experiments at different maturity stages.

YOLO11-Pose	CDC	C3H	New-Neck	P (%)	R (%)	mAP@50 (%)	mAP@50:95 (%)	Params (M)	FPS
✓				86.4	86.4	90.9	73.6	2.7	65.2
✓	✓			87.2	86.9	90.8	75.4	2.6	64.3
✓		✓		86.7	88.5	91.1	75.4	1.8	48.4
✓			✓	86.5	87.8	91.6	73.9	1.9	68.1
✓	✓		✓	87.1	87.8	91.1	76.7	1.9	65.8
✓	✓	✓		86.3	88.8	91.1	77.3	2.6	40.7
✓		✓	✓	86.6	87.8	91.0	75.6	1.9	53.3
✓	✓	✓	✓	87.3	88.0	91.8	78.6	1.9	71.6

**Table 2:** Ablation experiment results for key point detection in picking.

YOLO11-Pose	CDC	C3H	New-Neck	P (%)	R (%)	mAP@50 (%)	mAP@50:95 (%)	Params (M)	FPS
✓				81.1	85.4	88.3	79.0	2.7	65.2
✓	✓			85.0	83.3	88.2	82.1	2.6	64.3
✓		✓		84.0	85.8	89.1	81.5	1.8	48.4
✓			✓	81.7	85.2	88.4	81.4	1.9	68.1
✓	✓		✓	85.0	83.3	88.4	80.9	1.9	65.8
✓	✓	✓		84.8	85.4	89.0	82.1	2.6	40.7
✓		✓	✓	84.3	82.2	88.0	81.3	1.9	53.3
✓	✓	✓	✓	83.0	87.5	89.7	83.6	1.9	71.6

**Table 3:** Comparison of Experimental Results for Strawberry Recognition at Different Maturity Stages.

Model	P (%)	R (%)	mAP@50 (%)	mAP@50:95 (%)	FPS
YOLOv13-Pose	85.7	86.7	91.4	81.3	59.6
YOLO11-Pose	86.4	87.4	90.9	73.6	65.2
YOLOv10-Pose	83.6	86.3	91.7	74.4	85.7
YOLOv8-Pose	85.1	87.9	90.9	73.6	74.8
DHN-YOLO	87.3	88	91.8	78.6	71.6

**Table 4:** Comparative Experimental Results for Key Point Detection in Harvesting.

Model	P (%)	R (%)	mAP@50 (%)	mAP@50:95 (%)	FPS
YOLOv13-Pose	81.8	84.5	87.5	77.2	59.6
YOLO11-Pose	82.1	85.4	88.3	79	65.2
YOLOv10-Pose	81.4	87.5	89.0	81.3	80.7
YOLOv8-Pose	84	83.2	87.8	74.1	74.8
DHN-YOLO	83.0	87.5	89.7	83.6	71.6

## Data Availability

The data used in this study mainly include ridge-type strawberry images at multiple maturity stages, along with corresponding annotation data and key picking point labels. The dataset is relatively large, and it was independently collected by our research team for the purpose of model training in this study. At present, the data have not been organized into a format suitable for public sharing. Therefore, the authors can provide the relevant data upon reasonable request, provided that the data will be used for legitimate academic research purposes, to support verification and further study.
